# Supplementary material for: Safety and Effectiveness of an Exercise-Based Telerehabilitation Program in Myalgic Encephalomyelitis and Post COVID Syndrome: Protocol for a Randomized Controlled Clinical Trial
Source: Healthcare (Basel). 2025 Nov 26;13(23):3062. doi: 10.3390/healthcare13233062 (PMC12691739; doi:10.3390/healthcare13233062)
Supplement: Supplementary file 1 [file healthcare-13-03062-s001.zip › S1. Randomization script.pdf]

S1 FILE. RANDOMIZATION SCRIPT

"Mindful Exercise vs. Conventional Exercise vs. Usual Care"

==

Software: R (version ≥ 4.0)

Description:

This script reproduces the randomization procedure used in the trial, which assigned 147 participants to three study arms (1:1:1 ratio) in three consecutive waves (n = 50, 49, and 48). Each wave was randomized independently to maintain approximate balance (±1 participant per group) while preserving allocation concealment.

--- Note on allocation concealment ---

The actual randomization sequence generated from this script was handled by an independent collaborator not involved in participant recruitment. Sequentially numbered, opaque, sealed envelopes (SNOSE method) were used to conceal allocation until assignment. The randomization seed and this code are shared here to ensure transparency and reproducibility.

This script reproduces the algorithm used to generate the randomization lists in the trial.

The actual sequence file used during recruitment is not included to maintain allocation concealment.

Re-running the script with the specified seed (set.seed(20250922)) will reproduce the exact same sequence.

==

```
# --- Set reproducible seed (for transparency; may be replaced before recruitment) ---
set.seed(20250922)

# --- Define group labels and wave sizes ---
groups <- c("Mindful Exercise", "Conventional Exercise", "Usual Care")
wave_sizes <- c(50, 49, 48)   # Three consecutive recruitment waves

# --- Function to generate a balanced randomization list per wave ---
rand_per_wave <- function(n, groups) {
  k <- length(groups)
  base   <- rep(groups, each = n %/% k)  # equal allocation floor(n/k)
  extras <- sample(groups, n %% k)       # distribute remainders (0, 1, or 2)
```

```
  allocation <- sample(c(base, extras))  # random shuffle
  data.frame(Treatment = allocation, stringsAsFactors = FALSE)
}

# --- Generate randomization lists for each wave ---
lists <- list()
total <- 0
for (i in seq_along(wave_sizes)) {
  n_i <- wave_sizes[i]
  tmp <- rand_per_wave(n_i, groups)
  tmp$Wave <- paste0("Wave ", i)
  tmp$Order_in_wave <- seq_len(n_i)
  tmp$Participant_ID <- sprintf("R%03d", total + seq_len(n_i))
  lists[[i]] <- tmp[, c("Participant_ID", "Wave", "Order_in_wave", "Treatment")]
  total <- total + n_i
}

# --- Combine all waves into a single master table (not exported here) ---
randomization_master <- do.call(rbind, lists)
randomization_master$Global_order <- seq_len(nrow(randomization_master))
randomization_master <- randomization_master[,
  c("Global_order", "Participant_ID", "Wave", "Order_in_wave", "Treatment")]

# --- (Optional) Check distribution balance per wave ---
cat("\nBalance per wave:\n")
for (i in seq_along(lists)) {
  cat(paste0("  Wave ", i, " (n=", nrow(lists[[i]]), "):\n"))
  print(table(lists[[i]]$Treatment))
}
cat("\nTotal (n=", nrow(randomization_master), "):\n", sep = "")
print(table(randomization_master$Treatment))
```
