# Supplementary material for: Safety and Effectiveness of an Exercise-Based Telerehabilitation Program in Myalgic Encephalomyelitis and Post COVID Syndrome: Protocol for a Randomized Controlled Clinical Trial
Source: Healthcare (Basel). 2025 Nov 26;13(23):3062. doi: 10.3390/healthcare13233062 (PMC12691739; doi:10.3390/healthcare13233062)
Supplement: Supplementary file 1 [file healthcare-13-03062-s001.zip › S2. Detailed interventions description.pdf]

S2 FILE. DETAILED INTERVENTION DESCRIPTIONS

Safety notes (apply to all groups)

- Stop rules: stop/modify with dizziness, palpitations, unusual breathlessness, pressure-type headache, or any early signs suggestive of post-exertional malaise.
- Home safety: stable footwear or barefoot, clear floor space, sturdy chair/wall support.

Universal adaptation notes (apply to all groups)

- Symptom-contingent dosing: There is not a fixed intensity target. Adjust it depending on actual energy levels and keep intensity very light to light (2/3 Borg scale); use the talk test (able to speak in full sentences).
- HRV as trend only: Welltory metrics can inform decisions but do not replace symptom feedback.
- Rest as needed: extend rests if fatigue or dyspnoea rises.
- Progression: no automatic increases; only consider adding volume after 3 weeks practising with stable symptoms.
- Personalisation: participants choose the exercise adaptation, execution times, and repetitions to their medical context and clinician guidance. Position options: standing ↔ chair-supported ↔ seated/recumbent based on the day.
- Recovery first: priority to sleep regularity, hydration, nutrition, and stress-reduction behaviours.

Triggers to modify the intervention, pause sessions or discontinue the allocated intervention

Triggers to modify the intervention (dose titration, switching chair/standing options, longer rests, lower frequency or duration):

- Suffering an increase over 2/10 or reaching 7/10 in the Numeric Rating Scale for Fatigue or Pain related to sessions.
- Other non-serious adverse events judged to be related to sessions.
- Transient mild flare triggered by non-study related causes.
- Participant-reported excessive burden or two consecutive sessions aborted due to symptoms.

Process after AEs or safety concerns. We will offer a one-to-one safety videocall to review symptoms and adjust the plan.

Triggers to temporarily pause sessions:

- Intercurrent medical condition not related to the trial requiring short-term rest.

Triggers to discontinue the allocated intervention:

- Suffering an increase over 5/10 or reaching 9/10 in the Numeric Rating Scale for Fatigue or Pain related to sessions.
- Other serious adverse events or clinically significant deterioration judged related to the intervention.
- New medical diagnosis or condition that contraindicates exercise (per treating physician).
- Participant requests to stop the program.
- Start of another structured rehabilitation program or treatment that conflicts with the trial.

Mindful and conscious movement-based exercise (MBE) group:

EXERCISE

3 classes (to be repeated 4 times cyclically)

- *Class parts:*

5’ Pranayama (breathing exercises)

25’ Conscious movement (Chair Yoga) +5’ each month

5’ Yoga Nidra (mindful meditation) → proprioception exercises

- *Class themes:*

Full body (Integrated Movement)

Arms (Upper-body Strength)

Legs (Lower-body Strength)

- *Class organization:*

S1, S4, S7, S10: Class 1

S2, S5, S8, S11: Class 2

S3, S6, S9, S12: Class 3

—

Class 1: (Full body)

Time – Section – Exercise description

- *5’ – Pranayama*

• Breath and mindful observation: bring attention to the movement of the breath and the state of the body.

• Interoception exercises (1 + 2)

- *25’ – Movement*

• (x2) Sun Salutations A on the chair (Surya Namaskar A)

o Tadasana: Stand. Toes well rooted, crown upward, arms alongside the torso.

o Urdhva Hastasana: Inhale, lift arms overhead, gaze to the thumbs.

o Uttanasana: Exhale, hands to the floor, forehead to knees.

o Ardha Uttanasana: Inhale, hands to the chair, lengthen the spine, open chest and lift chin.

o Phalakasana (Plank): Exhale, step the feet back. Shoulders over wrists.

o Adho Mukha Svanasana: Exhale, hips up and back, gaze to the ankles. Hold for 5 breaths.

o Ardha Uttanasana: Inhale, take two big steps forward. Lengthen the spine.

o Uttanasana: Exhale, hands to the floor, forehead to knees.

o Urdhva Hastasana: Inhale, lift arms overhead, gaze to the thumbs.

o Tadasana: Exhale, hands by the sides of the body.

• (x1) Sun Salutations B (Surya Namaskar B)

Same as above but instead of Urdhva Hastasana, do Utkatasana on the inhale; and when reaching Adho Mukha Svanasana, add:

o Virabhadrasana I: Inhale, step one foot forward, bend the front knee and reach hands to the ceiling. Gaze between the palms.

o Repeat the steps from Phalakasana to Adho Mukha Svanasana for the other leg.

o After both sides, hold Adho Mukha Svanasana for 5 breaths before finishing the Sun Salutation.

Repeat on the other side.

Floor sequence (5 breaths each)

• Dandasana

• Paschimottanasana

• Janu Sirsasana A

• Ardha Matsyendrasana

• Baddha Konasana B

- *5' – Meditation / Nidra*

• Savasana

—

Class 2: (Arms)

Time – Section – Exercise description

- *5' – Pranayama*

• Breath and mindful observation

• Interoception exercises (1 + 4)

- *25' – Movement*

• (x1) Sun Salutations A on the chair

• (x1) Sun Salutations B on the chair

Standing sequence

• Raise and lower arms while seated on the chair

• Cactus arms with active trunk rotation

• Shoulder mobility with internal and external rotation while seated on the chair

• Press-ups on the floor or seated (V: knees down; hands on the chair; or standing with hands on a wall)

Floor sequence

• Dandasana

• Paschimottanasana

• Gomukhasana (V: bottom leg extended; use a strap/belt to hold hands behind the back)

- *5' – Meditation / Nidra*

• Savasana

—

Class 3: (Legs)

Time – Section – Exercise description

- *5' – Pranayama*

• Breath and mindful observation

• Interoception exercises (1 + 5)

- *25' – Movement*

• (x2) Sun Salutations A on the chair

• (x1) Sun Salutations B on the chair

Standing sequence:

• Squats

• Virabhadrasana I: Bend and extend the front knee

• Marjaryasana – Bitilasana: Alternate spinal flexion and extension in tabletop position or sitting on the chair.

Floor sequence

• Glute bridge

• Dandasana

• Paschimottanasana

• Upavistha Konasana A

- *5' – Meditation / Nidra*

• Savasana

Interoception exercises:

1. *Inhale* — close the eyes / *Exhale* — open the eyes.

2. *Inhale* — gently lift the chin and sternum towards the sky, opening the chest and anterior chain / *Exhale* — let the head and jaw drop, fully relaxing and opening the mouth.

3. Close the eyes. *Inhale* — move the (closed) eyes to the left / *Exhale* — move the eyes to the right. Repeat for several breathing cycles, then swap the pairings (left on exhale, right on inhale) and repeat the same number of cycles.

4. *Inhale* — press the feet into the floor and make a light chin tuck, lengthening through the crown of the head / *Exhale* — release everything, relaxing neck and legs.

5. Hands resting palms-down on the thighs. *Inhale* — supinate, turning the palms up / *Exhale* — extend the fingers, opening the palms / On the empty (after exhale) — pronate, returning the palms to the thighs.

6. *Inhale* — raise one arm forward / *Exhale* — move the arm out to the side, aligning it with the rib cage in the scapular plane / *Inhale* — turn the palm over and draw the scapula towards the rib cage (wrist supination, shoulder external rotation) / *Exhale* — open the palm, extend the fingers, and turn the head to the opposite side of the extended arm / *Inhale* — maintain the posture / *Exhale* — relax everything.

7. Close the eyes. *Inhale* — set the intention to let the eyeballs sink back in their sockets towards the back of the head / *Exhale* — change the intention to let the eyeballs move forward in their sockets, directing the gaze towards the front.

EDUCATION

- *Class 1. Pacing to prevent PEM*

Overview of pacing as a symptom-contingent strategy to prevent post-exertional malaise (PEM). Participants learn to budget energy across essential, important, and optional tasks; to plan rest in advance; and to use "movement snacks" rather than continuous sessions. Practical heuristics (talk test, RPE very-light to light) are paired with heart rate variability (HRV) trends from the Welltory app as a supportive daily reference (not diagnostic) to spot recovery/overload patterns. We emphasize flexible boundaries (good-day rules, bad-day rules), pre-commitment to stop criteria, and post-activity symptom logging.

- *Class 2. Awareness and conscious movement (intro to mindfulness)*

Defines bodily awareness, interoception, and mindful movement. We link awareness practices to safer pacing (earlier recognition of "yellow flags" like subtle cognitive fog or orthostatic cues) and to co-regulation of arousal. Short practices include breath-anchored body scans, slow range-of-comfort movements, and micro-pauses between repetitions to detect early fatigue. Brief introduction to mindfulness attitudes (non-judgement, curiosity, patience) to reduce over-pushing driven by frustration or fear of loss of fitness.

- *Class 3. Introduction to pain neuroscience (POBTE-based)*

Accessible education on why pain can persist and how meaning, context, and protective learning shape pain. We connect this perspective to movement experimentation within safety boundaries, graded exposure to feared but tolerable motions, and language that reduces threat. Public, open resources are signposted for self-education.

- *Class 4. Attention as a therapeutic tool*

Differentiates bottom-up bodily signals from top-down attention shaping. We train "attentional framing": narrow vs. open attention, and purposeful redirection toward task-relevant cues (breath, contact points, range-of-comfort) to avoid catastrophizing spirals. Practical drills: 90-

second focused-attention sets during movement; labelling sensations with neutral language; and short grounding sequences to reset when early warning signs appear.

- *Class 5. The autonomic nervous system (ANS)*

Brief primer on sympathetic/parasympathetic balance and why the ANS is both an indicator and regulator of internal states. We connect sleep regularity, light exposure, hydration, and meal timing with autonomic tone. HRV patterns (via Welltory) are discussed as trends that may reflect recovery, guiding day-to-day modulation of activity intensity and the scheduling of rest blocks.

- *Class 6. Syndromic pathophysiology (concise overview)*

High-level, non-technical overview of current hypotheses (e.g., post-infectious immune dysregulation, autonomic dysfunction, small-fibre changes, microcirculatory issues). Emphasis on heterogeneity of aetiologies and trajectories, normalizing symptom variability and reinforcing the need for individualized pacing rather than fixed progressions.

- *Class 7. Hypervigilance and perception*

Explains how heightened threat monitoring can amplify fatigue, pain, dyspnea, or palpitations. We teach "aware but not alarmed": noticing signals, checking context, and responding proportionally. Skills include: decentering thoughts, reframing "must finish" to "can pause," and using brief sensory anchors to prevent escalation without suppressing valid warning signs.

- *Class 8. Orthostatic intolerance (OI) strategies*

What orthostatic stress is and why it matters in daily life. Practical measures include position transitions in stages, legs-elevated pauses, cooling strategies, avoiding prolonged standing, and, where appropriate per prior medical advice, the use of compression garments, fluids/salt, and small frequent meals. We also introduce recumbent or semi-recumbent movement options and counter maneuvers when light-headedness appears.

- *Class 9. Digestive health and the gut–brain axis*

Education on how meal composition, timing, and portion size may influence symptoms (e.g., post-prandial fatigue, orthostatic worsening). Participants reflect on individual food triggers/tolerances, hydration, and gentle routines that support regularity. No diet is prescribed; the goal is symptom-informed experimentation within personal and medical constraints.

- *Class 10. Habits and adaptation*

The body is a learning system: consistent, low-noise inputs tend to stabilize function better than sporadic high-intensity efforts. We design tiny, repeatable routines (sleep–wake regularity, wind-down cues, brief movement snacks) and link them to existing anchors in the day. We emphasize systems over goals, celebrating adherence to safe patterns rather than volume totals.

- *Class 11. Supplementation: Potential benefits*

Educational module on different supplements like Q10, Zink, Magnesium and phosphocreatine, and their potential relevance to perceived energy. We review forms, expected effects, and typical contraindication screens. The trial does not prescribe any of these supplements; participants are advised to consult their clinician before any supplement use. Emphasis remains on behavioural energy management as the core strategy.

- *Class 12. Participant-selected topic (review/extension)*

Facilitated Q&A and deepening of requested themes (e.g., refining pacing plans, troubleshooting OI routines, refining mindful movement sequences).

Aerobic/strength low-intensity exercise group:

EXERCISE

Baseline sequencing

- *Warm-up (10 min; 1 min each): joint mobility + gentle stretches*

• *Wrist circles → forearm stretch*: slow circles (each way), then extend one arm, palm up, gently pull fingers back.

• *Ankles & knees*: ankle circles (each way); marching in place to mobilize knees.

• *Trunk rotations & lateral flexion*: hands across chest, rotate right/left (each side); slide one hand down the thigh for a side-bend.

• *Shoulders & elbows*: shoulder rolls forward/back (each side), elbow bends/extends.

• *Neck mobility*: slow rotations (each way), lateral tilt holds.

• *Inter-scapular activation*: standing, reach long through fists then pinch shoulder blades together.

- *Low-intensity strengthening (10 min)*

Perform 30 s work / 30 s rest; complete the 5 movements once or twice based on symptoms.

• *Body-weight Squat*

Set-up: feet hip-to-shoulder width, toes slightly out, chest tall.

Action: inhale to sit back as if to a chair, knees track over mid-foot; exhale to stand.

Cues: range-of-comfort; keep heels down. Regression: sit-to-stand from chair. Rest: 30–60 s.

• *Lateral Squat*

Wide stance; shift hips over one heel, other leg stays long; push the floor away to return.

Regression: reduce depth or hold onto a chair.

• *Chest Press (wall push-up)*

Hands on wall at chest height, body in straight line; inhale lowering, exhale pressing away.

Regression: step closer to wall; Progression: hands on bench/chair (incline push-up).

• *Front Plank*

Forearms on floor (or on the wall for incline), elbows under shoulders; ribs tucked, glutes lightly on.

Breathe quietly; stop early if shaking or breath-holding.

• *Heel Raise*

Feet hip width, rise onto balls of feet; brief pause at top, lower slowly (2–3 s).

Regression: hold chair; Progression: single-leg supported.

- *Low-intensity aerobic-functional (10 min)*

Perform 20 s work / 40–60 s rest; complete the 5 movements once or twice based on symptoms.

• *Adapted Jumping Jacks*: step one foot out while sweeping arms overhead; no jumping.

• *Arm Circles*: stand tall, circle forward 10 s, backward 10 s.

• *Knee Lifts*: march in place, knees to comfortable height, arms relaxed.

• *Push–Pulls*: alternating light punches forward then pull elbows back, smooth rhythm.

• *Box Steps*: step forward–forward–back–back inside an imaginary square.

Intensity anchor, keep the talk test easy (able to speak full sentences; RPE ≈ 2–3/10).

- *Cool-down & stretches (5 min)*

• Paced breathing (about 20 s): nasal inhale, longer exhale.

• Posterior chain + spinal flexion: seated or standing, fold gently with support (20–30 s).

• Lower limb stretches: calves (step stance, rear heel down), quadriceps (heel to chair or wall support), adductors (wide stance, lean to one side).

• Glute & posterior chain: figure-4 seated stretch (15–20 s/side); gentle standing forward fold with soft knees.

• Shoulder & neck rotations + lateral neck stretch: small circles, then ear-to-shoulder (15–20 s/side).

• Lie down or semi-recline for the remaining time to return towards resting heart rate.

Floor/recumbent-friendly option (for days with orthostatic symptoms)

- *Warm-up (10 min; 1 min each)*

Supine ankle pumps, knee fall-outs, pelvic tilts, shoulder blade clocks, neck nods, side-lying trunk rotation.

Gentle stretches: calves (using strap/towel), hamstrings (knee bent), adductors (butterfly), glutes (hip adduction).

- *Low-intensity strengthening (20 min)*

Perform 30 s work / 1 min rest; complete the 6 movements from one to twice based on symptoms.

• Glute bridge

• Weighted chest press

• Hip flexion leg raises

• Weighted shoulder flexion

• Weighted triceps press

• Recumbent heel raise with bended knees

- *Cool-down (5 min)*

Paced breathing (longer exhale), hamstring/posterior chain stretch (supine with strap), spinal rotation (supine knees together), shoulder rolls/neck lateral stretch seated, then supine rest with legs elevated if helpful.

EDUCATION

- *Class 1. Pacing to prevent PEM*

Overview of pacing as a symptom-contingent strategy to prevent post-exertional malaise (PEM). Participants learn to budget energy across essential, important, and optional tasks; to plan rest in advance; and to use "movement snacks" rather than continuous sessions. Practical heuristics (talk test, RPE very-light to light) are paired with heart rate variability (HRV) trends from the Welltory app as a supportive daily reference (not diagnostic) to spot recovery/overload patterns. We emphasize flexible boundaries (good-day rules, bad-day rules), pre-commitment to stop criteria, and post-activity symptom logging.

- *Class 2. Health benefits of exercise*

Overview of general benefits (metabolic, cardiovascular, mood, sleep) while clarifying that in post-infectious conditions the dose–response can differ. The focus is on finding an individually tolerable window (very-light to light), not on progressive overload. We reframe success as consistency without flare-ups.

- *Class 3. Introduction to pain neuroscience (POBTE-based)*

Accessible education on why pain can persist and how meaning, context, and protective learning shape pain. We connect this perspective to movement experimentation within safety boundaries, graded exposure to feared but tolerable motions, and language that reduces threat. Public, open resources are signposted for self-education.

- *Class 4. Low-intensity exercise: a paradigm shift*

Rationale for favouring low intensity and avoiding fixed, automatic weekly increments. We discuss potential drawbacks of rigid graded escalation in people prone to PEM and propose alternative progress markers (symptom stability, recovery time, perceived control). Practical menu: very-light aerobic options (e.g., slow recumbent cycling, gentle walking intervals), mobility, and low-load strengthening with long rests.

- *Class 5. The autonomic nervous system (ANS)*

Brief primer on sympathetic/parasympathetic balance and why the ANS is both an indicator and regulator of internal states. We connect sleep regularity, light exposure, hydration, and meal timing with autonomic tone. HRV patterns (via Welltory) are discussed as trends that may reflect recovery, guiding day-to-day modulation of activity intensity and the scheduling of rest blocks.

- *Class 6. Syndromic pathophysiology (concise overview)*

High-level, non-technical overview of current hypotheses (e.g., post-infectious immune dysregulation, autonomic dysfunction, small-fibre changes, microcirculatory issues). Emphasis

on heterogeneity of aetiologies and trajectories, normalizing symptom variability and reinforcing the need for individualized pacing rather than fixed progressions.

- *Class 7. Deconditioning and overexertion*

Explains why maintaining some level of activity matters within the safe window, while also avoiding boom–bust cycles. We teach “floor not ceiling”: establish a dependable minimum on most days, add optional extras only when recovery is solid, and step back promptly at early warning signs.

- *Class 8. Orthostatic intolerance (OI) strategies*

What orthostatic stress is and why it matters in daily life. Practical measures include position transitions in stages, legs-elevated pauses, cooling strategies, avoiding prolonged standing, and, where appropriate per prior medical advice, the use of compression garments, fluids/salt, and small frequent meals. We also introduce recumbent or semi-recumbent movement options and counter maneuvers when light-headedness appears.

- *Class 9. Digestive health and the gut–brain axis*

Education on how meal composition, timing, and portion size may influence symptoms (e.g., post-prandial fatigue, orthostatic worsening). Participants reflect on individual food triggers/tolerances, hydration, and gentle routines that support regularity. No diet is prescribed; the goal is symptom-informed experimentation within personal and medical constraints.

- *Class 10. Habits and adaptation*

The body is a learning system: consistent, low-noise inputs tend to stabilize function better than sporadic high-intensity efforts. We design tiny, repeatable routines (sleep–wake regularity, wind-down cues, brief movement snacks) and link them to existing anchors in the day. We emphasize systems over goals, celebrating adherence to safe patterns rather than volume totals.

- *Class 11. Supplementation: Potential benefits*

Educational module on different supplements like Q10, Zink, Magnesium and phosphocreatine, and their potential relevance to perceived energy. We review forms, expected effects, and typical contraindication screens. The trial does not prescribe any of these supplements; participants are advised to consult their clinician before any supplement use. Emphasis remains on behavioural energy management as the core strategy.

- *Class 12. Participant-selected topic (review/extension)*

Facilitated Q&A and deepening of requested themes (e.g., refining pacing plans, troubleshooting OI routines, refining mindful movement sequences).
